# Age Norms and the Spirit of Capitalism

**DOI:** 10.1093/geroni/igab046.2306

**Published:** 2021-12-17

**Authors:** Jeremiah Morelock

**Affiliations:** Boston College, Woburn, Massachusetts, United States

## Abstract

Adulthood is often associated with hard work, in contrast to childhood and later life, which are associated with play, education, and leisure. Yet the work-fixated sense of adulthood is about more than just age norms. Like any such ethos, it is situated in socioeconomic history. Workers are forced to work hard, the work ethic framing their exploitation within an aura of moral righteousness. According to Weber the normative weight commonly associated with ‘hard work’ derives from the advent of Protestantism in the late middle ages. Weber says that this new worldview birthed the ‘spirit of capitalism,’ and set the stage for the modern world to take shape. In the seventeenth century—hence roughly coinciding with mercantilism and the Reformation—was the invention of the modern concept of childhood, i.e. the radical division of childhood from adulthood. This period also inaugurated the European Enlightenment, where reason was elevated as a supremely honorable aspect of humanity, in many ways as a new source of this-worldly pseudo-salvation. ‘Adulthood’ was infused with these values—the ideal [male] adult is rational, responsible, hard-working, self-sufficient, and financially secure. It was adulthood, more than and in contrast to other times of life (e.g., childhood and later life), that absorbed and normalized the new economic and cultural trends. The moral elevation of hard work, combined with the greater demarcation of adulthood in contrast to childhood and later life, set up children and older adults to take on a status of moral inferiority due to their exclusion from the working world.

